# Optimizing Aortic Arch Stent-Graft Performance Through Material Science: An Exploratory Study

**DOI:** 10.3390/ma18153592

**Published:** 2025-07-31

**Authors:** Xiaobing Liu, Linxuan Zhang, Zongchao Liu, Shuai Teng

**Affiliations:** 1 College of Transportation and Logistics, Guangzhou Railway Polytechnic, Guangzhou 511300, China; liuxiaobing@gtxy.edu.cn (X.L.); zhanglinxuan@gtxy.edu.cn (L.Z.); 2 School of Civil and Transportation Engineering, Guangzhou University, Guangzhou 510006, China; tengs@gzhu.edu.cn

**Keywords:** stent-graft, mechanical properties, material properties, aortic arch, finite element method

## Abstract

Thoracic endovascular aortic repair (TEVAR) for cardiovascular diseases often encounters complications that are closely linked to the mechanical properties of stent-grafts. Both the design and material properties influence device performance, but the specific impacts of material properties remain underexplored and poorly understood. This study aims to fill this gap by systematically investigating how material science can modulate stent-graft mechanics. Four types of bare nitinol stents combined with expanded polytetrafluoroethylene (e-PTFE) or polyethylene terephthalate (PET) grafts were modeled via finite element analysis, creating eight stent-graft configurations. Key mechanical properties—flexibility, crimpability, and fatigue performance—were evaluated to dissect material effects. The results revealed that nitinol’s properties significantly influenced all performance metrics, while PET grafts notably enhanced flexibility and fatigue life. No significant differences in equivalent stress were found between PET and e-PTFE grafts, and both had minimal impacts on radial force. This work underscores the potential of material science-driven optimization to enhance stent-graft performance for improved clinical outcomes.

## 1. Introduction

Endovascular aortic repair (EVAR) with a stent-graft (SG) to treat vascular diseases has the advantages of being minimally invasive and causing low mortality and morbidity compared with traditional open surgery [1,2,3]. However, the extreme curvatures and significant morphological differences of the aortic arch among patients demand superior device performance [4,5]. Clinical complications such as endoleak, device migration, new entry, fracture, and retrograde dissection related to the mechanical properties of SGs are frequently reported [6,7,8,9,10].

Flexibility, crimpability, and fatigue life, commonly considered to be important mechanical properties of devices, have been proved to be significant factors inducing the aforementioned clinical complications [10,11,12,13]. Adequate flexibility enables the compressed stent to be easily transferred into tortuous arteries, and it also helps the expanded stent to follow the vessel’s contour easily and produce less distortion at the stent-vessel interface [14,15]. One aspect of crimpability is the radial force, which can recover the normal diameter of narrowed vessels and anchor the device to the vessel. Vessel restenosis and device migration are induced by inadequate radial force, but excessive radial force can result in vessel damage [16,17,18]. In addition, a lack of flexibility and device migration can both result in endoleak [13,19]. Another aspect of crimpability is the compressive strength, which reflects the safety of SGs during large-deformation compression before entering the vessels. Static fracture of SGs can be induced when the mechanical strength of the material is exceeded. Fatigue fracture can be induced by the cyclic blood pressure exerted upon the stent [20]. Nitinol stents have been widely used in thoracic endovascular aortic repair (TEVAR) for their outstanding super-elasticity, high flexibility, and shape memory [12]. Different with the conventional engineering materials, the fracture of nitinol stents is not stress-based but strain-based, and the oscillating strain amplitude is the main contributor to fatigue behavior [12].

Recent studies have made great progress in improving device performance by changing the structure design [19,21,22]. The geometric parameters of SGs that affect the flexibility, radial force, and fatigue performance have been determined, and a series of optimizations have been conducted on these parameters [21,23,24]. In addition, the stent design (such as different limbs and link configurations) also significantly influences the stent’s flexibility [25,26]. Nitinol is a thermo-mechanical coupled super-elastic material, and it is composed of certain proportions of nickel metal and titanium metal [27]. The material composition, temperature environment, and loading rate influence the material properties of nitinol [28], which in turn influence the mechanical properties of nitinol devices. In one study, the crimpability of nitinol stents with different materials was analyzed, and differences in the maximum crimping strain were obtained [29]. In another study, both the metal stent and the graft material were analyzed with regard to their influence on the performance of SGs. Two nitinol stents covered with a PET graft or an e-PTFE graft were analyzed to compare their crimpability, compliance, and fatigue life. One of the combinations showed a low safety factor and did not meet the requirement. The PET graft exhibited a great contribution in improving the fatigue life of the SG but decreased the compliance [30]. These two polymers are the most extensively utilized in commercial nitinol stent-grafts, with a wealth of long-term clinical data validating their efficacy. However, studies on the influence of material properties on the performance of stents are very rare, and the potential and feasibility of improving the mechanical properties of SGs by adjusting their material properties are still unclear.

Numerical simulation is an efficient and popular tool to investigate the biomechanical behaviors of SGs [31]. It provides intuitive and visual results and is convenient for conducting a large number of analyses. This study explores the potential for enhancing SG performance by optimizing the material selection and microstructure. It first summarizes four nitinols frequently used in the existing literature, adopting PET and e-PTFE as grafts. A total of eight finite element models of SGs are constructed. The flexibility, crimpability, and fatigue performance of these models are analyzed, and factors causing performance differences among them are discussed, aiming to provide valuable references for optimizing SG performance.

## 2. Materials and Methods

### 2.1. Geometry and Materials

**Figure 1:**
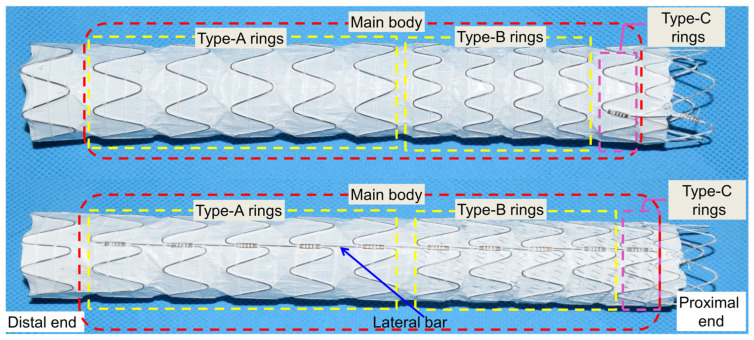
Commercial stent-graft and geometric configuration of commercial descending aortic stent-graft [32].

The commercial descending aortic SG (Lifetech Scientific Corporation, Shenzhen, China) adopted in this work is shown in Figure 1. The asymmetric main body consisted of three kinds of rings with different heights, classified as Type-A, Type-B, and Type-C rings. All the rings were chained with lateral bars. Type-A and Type-C rings included seven struts with a height of 15 mm and nine struts with a height of 12 mm, respectively. The Type-B ring included a mix of four struts with a height of 14 mm and five struts with a height of 14 mm. To decrease the computational cost without affecting the results, three Type-A rings, four Type-B rings, and one Type-C ring were modeled in the finite element models. All the struts had the same radius of 1.2 mm. The diameters of the SG and stent wire were 36 mm and 0.55 mm, respectively. The bare metal stent was fully covered by a 0.1 mm thick and 150 mm long graft. The finite element model of the SG was established in Unigraphics NX (version 10.0, Siemens PLM Software, Munich, Germany) and analyzed in ABAQUS-6.14 (Dassault Systemes, Waltham, MA, USA, version 6.14-1).

**Figure 2:**
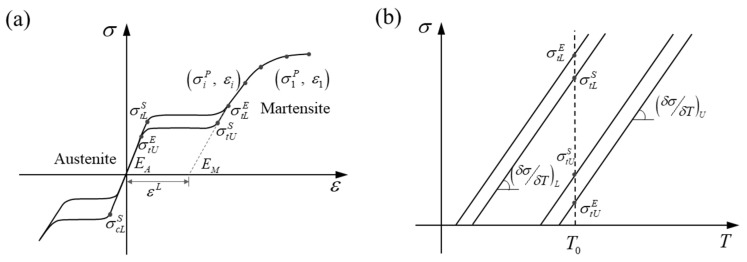
Constitutive relationship of nitinol [32]: (**a**) stress-strain curve; (**b**) stress-temperature curve.

The super-elasticity and shape memory of nitinol come from the transformation between austenite and martensite. With the incentive of an external load or decrease in temperature, the austenite-to-martensite transformation occurs when the start of transformation loading ($\sigma_{tL}^{s}$) is reached. The transformation part follows standard plasticity rules, where the stress remains stable but the strain increases quickly. The transformation is fully completed when transformation loading ($\sigma_{tL}^{e}$) stops and the crystal in nitinol has been converted from austenite to martensite. The stress-strain relation is linear until the plastic yield stress is reached ($\sigma_{1}^{p}$). The reverse martensite-to-austenite transformation occurs in the unloading process or with an increase in temperature. The start and end of transformation loading are expressed as $\sigma_{tU}^{s}$ and $\sigma_{tU}^{e}$, respectively. Nitinol can recover its predetermined shape along the unloading path; so, it is called a shape memory alloy. The strain increment is decomposed into a linear elastic part and a stress-induced transformation part ($\varepsilon_{L}$) [33]. Since the transformation strains are much larger compared to the typical elastic strains in a metal, nitinol is said to be super-elastic [12]. The constitutive relationship of nitinol is depicted in Figure 2, and four kinds of nitinol summarized from the literature are listed in Table 1. The nitinol material used in this study was modeled in ABAQUS as a user material (VUMAT), which was a thermo-mechanical coupled super-elastic model.

In this study, commonly used materials including e-PTFE and PET were employed to investigate their influence on device performance. Their material properties are listed in Table 2, and they were modeled with a linear elastic model.

In total, eight finite element models were established. They had the same structural parameters, and their bare stent and graft materials are listed in Table 3.

### 2.2. Mechanical Properties

#### 2.2.1. Bending Test

**Figure 3:**
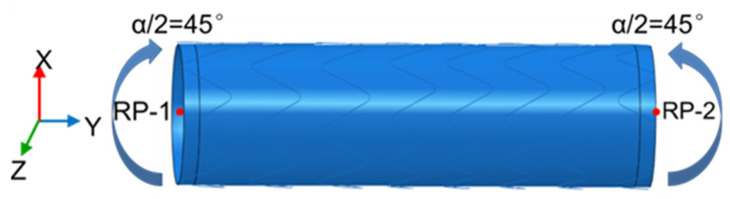
Bending test: boundary conditions and loads [19].

A bending test was carried out to analyze the flexibility of different models. Both ends of the SG were set as a rigid body, and a 45° rotation (around the Z-axis) was applied on each of the reference points (RP-1 and RP-2) (Figure 3). The rigid motions and rotations were constrained in all directions except the motion along the Y-axis (Figure 3). The mesh convergence was verified in previous work, and the SG was meshed with an element size of 0.6 mm [21]. Beam elements (B31) were used to model the metal stent for its advantage in the bending experiment, and shell elements (S4) were generated in the graft. The “bonding” algorithm was used to simulate the suture and prevent sliding between the graft and metal stent during the bending process. The self-contact algorithm was adopted to avoid the self-penetration of components. The stent surface was set as the master surface, and the graft surface was the slave surface. The analyses were executed in ABAQUS 6.14/Explicit. The ratio between global kinetic and strain energies was kept to a maximum of 10% in order to remain in a quasi-static state. The above settings were also used for the analysis of the following performance.

In this study, the flexibility was defined as follows:

(1) $$\delta_{F}=\frac{1}{{\mathrm{RM}}_{\max}}$$

where RM_max_ is the maximum reaction moment during the bending process.

#### 2.2.2. Crimping Test

**Figure 4:**
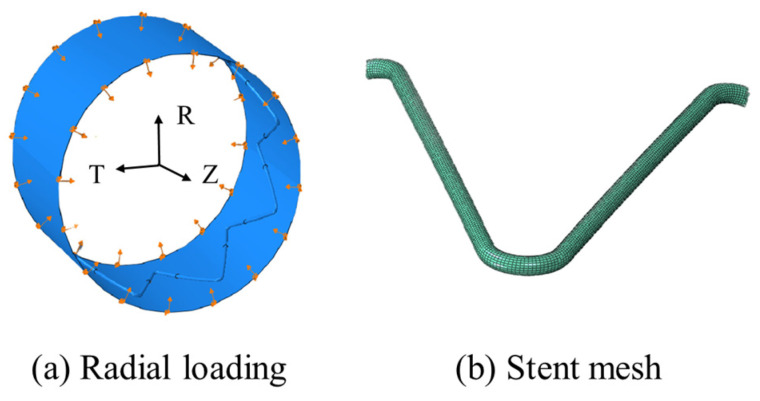
Radial compression and meshed stent (arrows in (**a**) mean radial force).

A 5 mm displacement was applied on the proximal stent ring via a crimping tool (a cylindrical rigid surface) in the radial direction to compare the radial forces among the models (Figure 4a). The radial force (*RF*) was defined as the sum of the normal contact force (*CNORMF*, output field in ABAQUS) of the nodes in the crimping tool, as RF=ΣCNORMF. A “hard” contact algorithm and a tangential penalty algorithm with a friction coefficient of 0.3 were used to enforce impermeable boundaries. The inner surface of the crimping tool was selected as the master surface, and the SG surface was set as the slave. The circumferential and axial motions of the SG ring were constrained via one node under a cylindrical coordinate system. The stent was meshed in HyperMesh (Altair Engineering Inc., Baltimore, MD, USA, version 2022), and the 8-node incompatible solid element (C3D8I) (Figure 4b) was adopted. The mesh sensitivity and density were verified in our previous research [19]. Finally, 22,700 and 153,000 elements were meshed in the stent and graft, respectively. The crimping tool was meshed with reduced surface elements (SF3M4R).

#### 2.2.3. Fatigue Loading

Due to their remarkable super-elasticity, shape memory, biocompatibility, and fatigue life, nitinol stents are becoming more popular in clinical applications [34]. Compared to conventional engineering materials, nitinol shows excellent fatigue properties at high strain levels. Recent studies have confirmed that nitinol stent fracture is not stress-based but strain-based. Furthermore, both the effects of mean and alternating strains contributing to the fatigue behavior of nitinol have been studied. It has been pointed out that the durability of a nitinol stent is determined by the alternate strain amplitude under periodic and long-term blood pressure [35]. In the fatigue analysis, arterial pressure from 50 mmHg (diastolic) to 150 mmHg (systolic) was applied to the inner surface of the SG, and the alternate strain amplitude was obtained as follows [12]:

(2) $$\varepsilon_{a}=0.5\left(\varepsilon_{s}-\varepsilon_{d}\right)$$

where $\varepsilon_{a}$ represents the alternate strain amplitude; $\varepsilon_{s}$ and $\varepsilon_{d}$ are the systolic and diastolic strains, respectively. Finally, the durability factor (DF) was obtained as follows:

(3) DF=εlimεa

where $\varepsilon_{\lim}$ is the fatigue limit of the nitinol stent under the periodic systolic and diastolic pressure, and it is 0.4%.

The contact algorithm and boundary conditions in this part were kept the same as those mentioned in Section 2.2.1 and Section 2.2.2, respectively.

## 3. Results and Discussion

### 3.1. Flexibility

**Figure 5:**
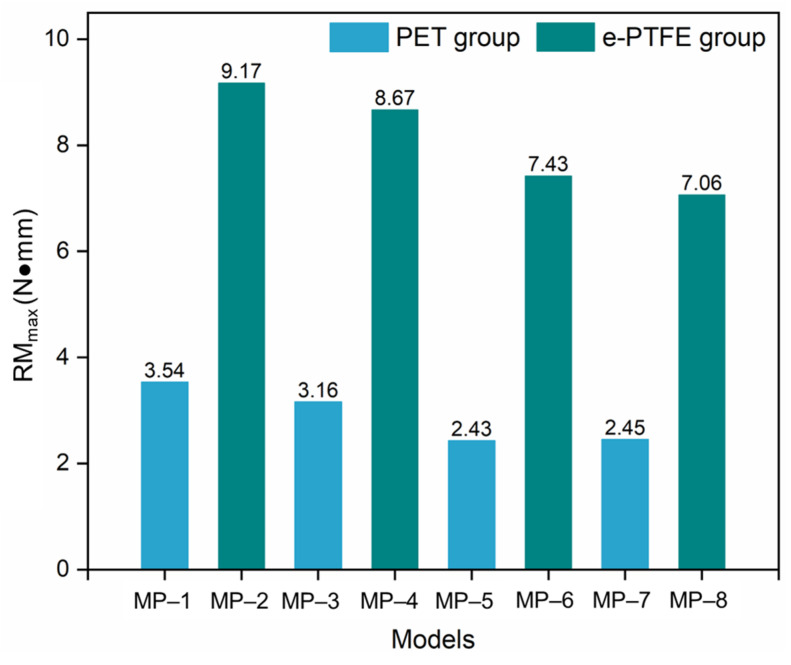
Differences among the maximum *RM* values of eight SG models (note: *p* > 0.05, not significant; 0.01 < *p* < 0.05, significant; *p* < 0.01, very significant).

The maximum reaction moment (RM_max_) of all eight models is shown in Figure 5. Figure 5 shows the significant influence (*p* < 0.01) of the graft material on SG flexibility, as the SGs in the PET group had much lower RM_max_ than those in the e-PTFE group. This indicated that the stents in the PET group had better flexibility than those in the e-PTFE group according to Equation (1). The RM_max_ in both groups was sorted in the same order, and the differences among the models were not significant (*p* > 0.05). The highest RM_max_ in both the PET and e-PTFE groups was achieved by the stents made of nitinol-1. This indicated that nitinol-1 was disadvantageous for achieving excellent flexibility. In addition, the stents made from nitinol-3 and nitinol-4 had the closest and lowest RM_max_, making them the best materials for improving flexibility.

**Figure 6:**
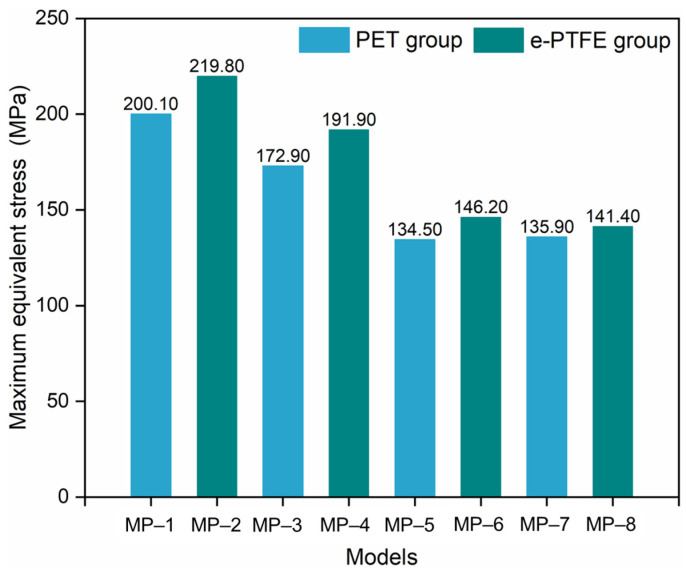
Maximum equivalent stress in stents with materials.

The maximum equivalent stress (Figure 6) in the metal stent could be used to illustrate the above results. The maximum equivalent stresses ranged from 134.50 MPa (MP-1) to 219.80 MPa (MP-2), and all of them were much lower than their start of transformation loadings ($\sigma_{tL}^{s}$). Therefore, the crystals of all the nitinol stents remained in an austenitic state after bending. The flexibility was determined by the material properties of austenitic nitinol, namely, *E_A_* and $\mu_{A}$. The *E_A_* values in descending order were as follows: nitinol-1 > nitinol-2 > nitinol-3 > nitinol-4; this order also represented the sorting of flexural rigidity. By comparing MP-5 and MP-7, MP-6, and MP-8, a difference was found in the flexibility of nitinol-3 and nitinol-4, which was caused by $\mu_{A}$. It was also found that the flexibility was dominated by *E_A_*.

Multiple linear regression (MLR) was used to establish the functional relationships between RM_max_ and the two parameters in the PET and e-PTFE groups. The fitting was performed based on normalized independent variables and dependent variables, and the result is shown in Equation (4):

(4) $$\left\{\begin{array}{l} f_{{\mathrm{PET},\mathrm{RM}}_{\max}}\left(E_{A},\mu_{A}\right)=0.08475+0.93826x_{1}-0.02123x_{2},{\;R}^{2}=0.987\\ f_{{e-\mathrm{PTFE},\mathrm{RM}}_{\max}}\left(E_{A},\mu_{A}\right)=0.55779+0.56775x_{1}-0.16829x_{2},{\;R}^{2}=0.990 \end{array}\right.,$$

where *x*_1_ and *x*_2_ represent *E_A_* and $\mu_{A}$, respectively. Their adjusted errors (*R*^2^) were 0.98664 and 0.98968. According to Equation (4), RM_max_ can be predicted from *E_A_* and $\mu_{A}$, and the flexibility decreases with *E_A_* and increases with $\mu_{A}$.

**Figure 7:**
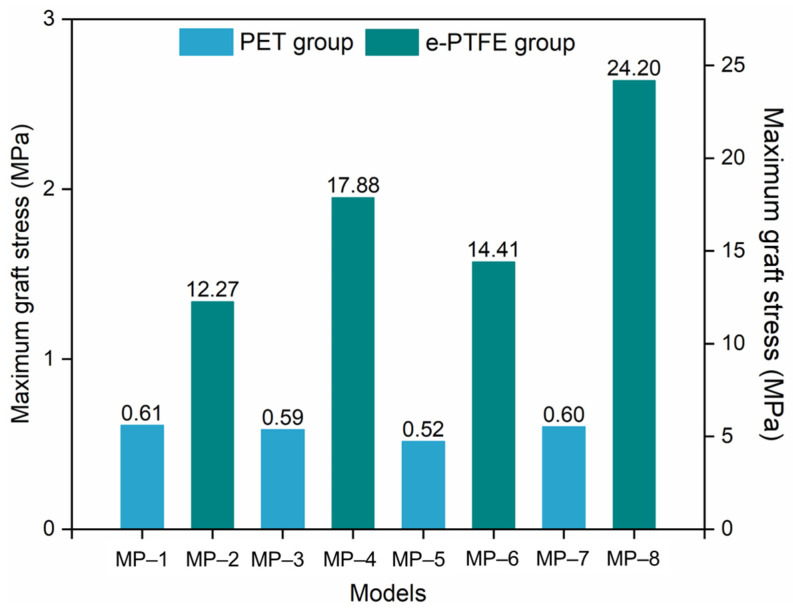
Maximum stress in the grafts (note: *p* > 0.05, not significant; 0.01 < *p* < 0.05, significant; *p* < 0.01, very significant).

The maximum graft stress is shown in Figure 7. The difference between the PET and e-PTFE groups was very significant because *p* < 0.01. The maximum stress in the PET graft ranged from 0.52 MPa to 0.61 MPa, which was much lower than that in the e-PTFE graft (from 12.27 MPa to 24.2 MPa). Indeed, the maximum stress of metal stents in the PET group was also lower than that of stents in the e-PTFE group (Figure 6). Therefore, the integral stiffness of the e-PTFE group was higher than that of the PET group. The similar graft stress of PET grafts illustrates that metal stent properties slightly affect PET grafts. Compared to the PET group, the compatible deformation capability between nitinol alloys and e-PTFE grafts is worse, because there is a significant difference in graft stress within the e-PTFE group. For MP-8, both the nitinol alloy and the graft were close to incompressible (*μ* = 0.46), with the stress in the graft being the highest. The lowest graft stresses were from the combinations of nitinol-1/PET and nitinol-1/e-PTFE. In the e-PTFE group, the maximum stress of each model exceeded the yield stress (Table 3, 6.6 MPa), which was not the case in the PET group. A tear risk exists for the SGs in the e-PTFE group.

The material properties listed in Table 1 and Table 2 may explain the above results. Compared with e-PTFE, PET has a lower elastic modulus, indicating lower flexural rigidity, but greater yield stress, indicating better elasticity. The equivalent stress and RM_max_ of SGs in both the PET and e-PTFE groups followed an identical rule (Figure 5 and Figure 6). The maximum equivalent stresses of all the models were much lower than their starting transformation stresses. Therefore, the material properties (including the elastic modulus and Poisson’s ratio) in the austenitic state of nitinol determine the flexibility of SGs. For instance, nitinol-1 stents were the most difficult to bend and exhibited the highest RM_max_ values among all models, which were accompanied by the stiffest flexural rigidity, and they had the worst flexibility. The stents made of nitinol-3 and nitinol-4 had similar flexibility for the same austenitic elastic modulus. The slight difference between them may have been induced by the different Poisson’s ratios (Table 1). Nitinol-4 was close to the incompressible state (referring to incompressible volume, where μ=0.5). The incompressible state induced horizontal deformation (perpendicular to the stent axis) and increased the bending difficulty. Therefore, the flexibilities of MP-7 and MP-8 were slightly worse than those of MP-5 and MP-6, respectively.

In addition, the stress fields further confirmed that the e-PTFE graft increased the bending resistance, as stents covered with e-PTFE grafts showed higher maximum stresses than did those covered with PET grafts.

### 3.2. Radial Force

**Figure 8:**
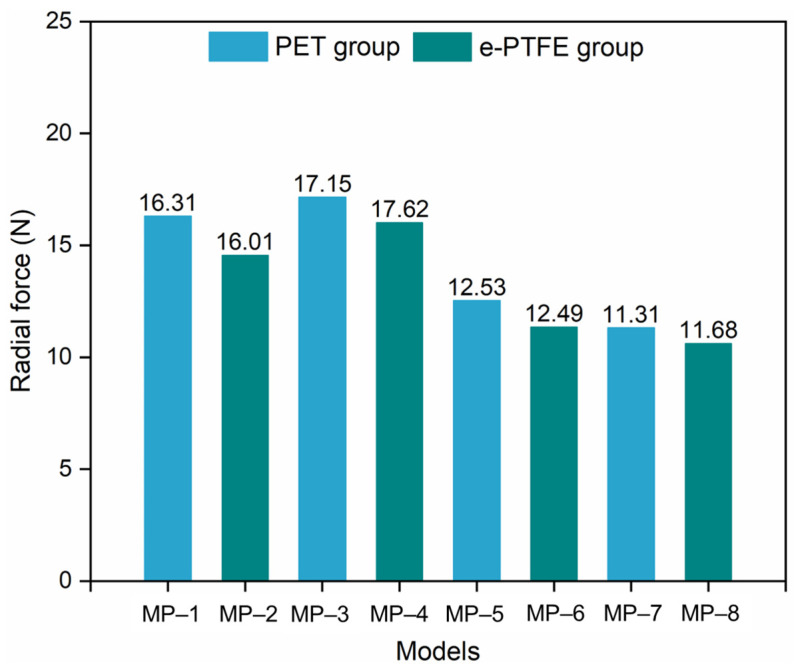
Radial force of different stent-grafts (note: *p* > 0.05, not significant; 0.01 < *p* < 0.05, significant; *p* < 0.01, very significant).

The *RF*s of the SGs with different materials are shown in Figure 8. There was no obvious difference between the *RF*s obtained from the same nitinol but different graft materials (*p* > 0.05). Without considering the graft, the difference among the stents made of different nitinols was distinct. The stent made of nitinol-2 exhibited the largest *RF* among all the nitinol stents. The *RF* of the nitinol-1 stent was slightly lower than that of the nitinol-2 stent, but it was much larger than those of the nitino-3 and nitinol-4 stents. In addition, nitinol-1 and nitinol-2 stents had similar *RF*s, while nitinol-3 and nitinol-4 stents had similar *RF*s.

**Figure 9:**
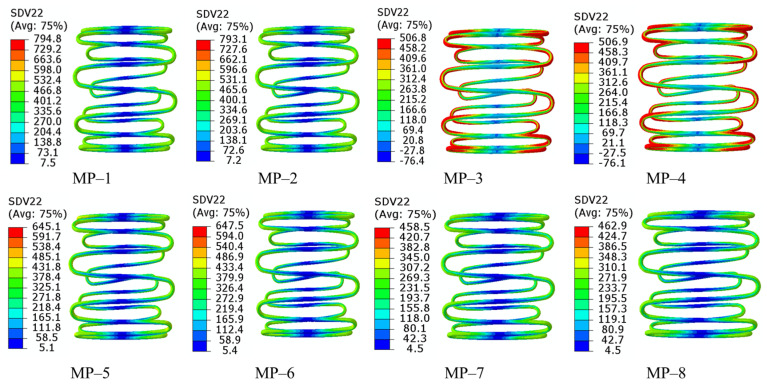
Equivalent stresses in different SGs under the compression state.

The equivalent stress fields after compression are shown in Figure 9. The stents made of the same nitinol had a similar stress distribution and maximum equivalent stress, regardless of the type of graft used. From this combined with the phenomena in Figure 8, it could be concluded that the effect of the graft on the crimpability of the SGs was negligible, and this conclusion agrees with the existing literature [30]. In MP-3 and MP-4, numerous points reached a maximum equivalent stress of around 506 MPa, whereas other models did not show this stress behavior. The largest maximum stress of 794 MPa was generated at MP-1, and the lowest (458 MPa) was generated at MP-7. It is important to point out that the transformation stresses of nitinol-2 and nitinol-4 listed in Table 1 were based on a reference temperature of 37 °C. However, the temperature set in the analyses was 22 °C. The starting transformation stresses of the two nitinols under 22 °C could be obtained by the following equation according to Figure 2b:

(5) $$\sigma_{T}=\sigma_{T_{0}}-\frac{\delta \sigma }{\delta T}\cdot \left(T_{0}-T\right)$$

where $\sigma_{T_{0}}$ is the known transformation stress at the reference temperature *T*_0_, *T* is an arbitrary temperature, and δσδT is the coefficient listed in Table 1. Finally, the starting transformation stresses were 502 MPa and 292 MPa, and the ending transformation stresses were 572 MPa and 327 MPa. Therefore, MP-3 and MP-4 remained in the transformation phase after compression, whereas the other models finished the transformation, and a portion of the nitinol crystal entered into a martensite state. The large deformation generated in MP-3 and MP-4 was caused by lower stress compared with other SGs. The crimpability of the stents made of nitinol-2 mainly relied on the properties of austenite, while that of the stents made of the other three nitinols was determined by the properties of both austenite and martensite.

Generally, the crimpability of SGs closely depends on the properties of nitinol. The nitinols used to make MP-1, MP-2, MP-5, MP-6, MP-7, and MP-8 remained in a mixed crystal state, and their crimpability (both radial force and stress) was determined by the properties in both crystal states. The *RF* and maximum stress obtained from the nitinol-1 stent were the largest due to its highest stiffness in both crystal states, and the difference between nitinol-3 and nitinol-4 stents mainly came from martensitic stiffness. Although the austenitic stiffness of nitinol-2 was lower than that of nitinol-1, the RFs of MP-3 and MP-4 were greater than those of MP-1 and MP-2, respectively. The most likely reason for this is that the transformation strain of nitinol-2 (0.063) is much larger than that of nitinol-1 (0.041). The deformation increases, while the stress remains at a stable value during the transformation phase. Therefore, the compressive stresses in MP-3 and MP-4 were much lower than those in MP-1 and MP-2 (Figure 9). The stress fields in MP-3 and MP-4 indicated that nitinol-2 did not enter the martensitic crystal state, and the stiffness was determined by the austenitic stiffness. However, a large portion of the crystal in nitinol-1 entered the martensitic state, and the martensitic stiffness of nitinol-1 decreased significantly compared with the austenitic stiffness of nitinol-1 and nitinol-2. This is why the RFs of MP-3 and MP-4 were slightly higher than those of MP-1 and MP-2, respectively.

### 3.3. Fatigue Performance

**Figure 10:**
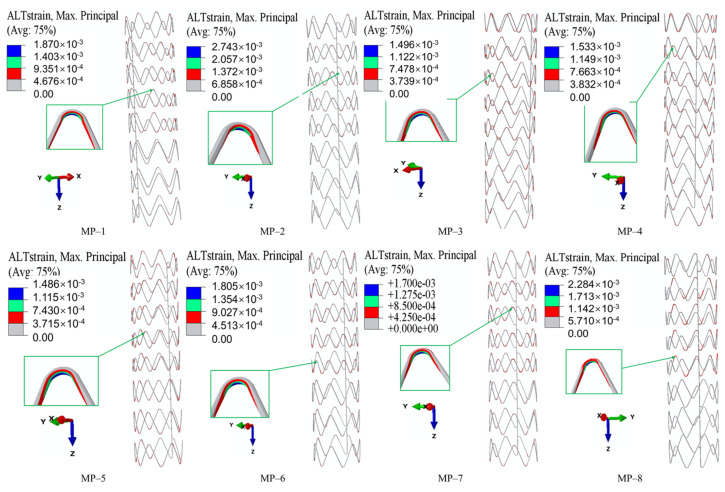
Maximum alternate strain and corresponding position.

**Figure 11:**
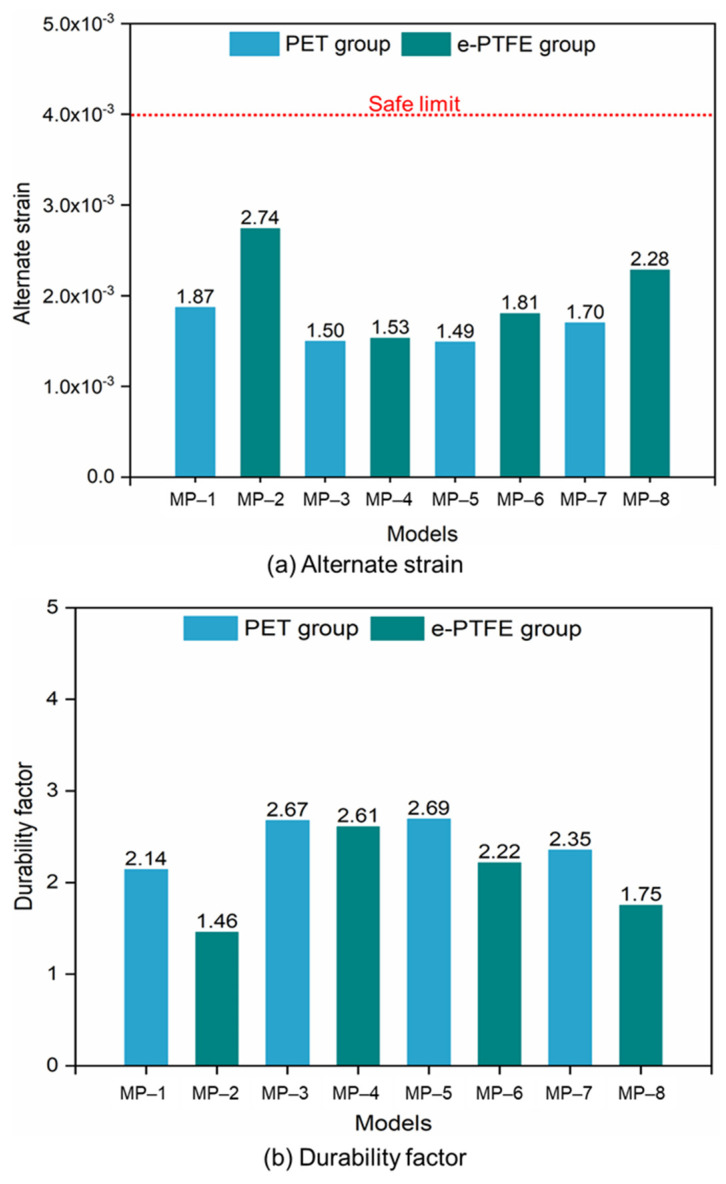
Fatigue performance of different SG models: (**a**) alternate strain; (**b**) durability factor (note: *p* > 0.05, not significant; 0.01 < *p* < 0.05, significant; *p* < 0.01, very significant).

The alternate strain fields representing the fatigue performance of SGs under long-term cyclic loading are shown in Figure 10. They were obtained by a Python (version 3.5) script based on the ABAQUS output results. The alternate strains at the strut crest were larger than those at the straight links. The maximum alternate strains of MP-1 and MP-8 were located at the crest of the higher strut in a Type-B ring, and the maximum alternate strains of other models were located at the crest of the shorter strut in a Type-B ring. Generally, the alternate strains in Type-A and Type-C rings were lower than those in Type-B rings. The maximum alternate strains of every model were lower than the limit (0.4%), and the largest and lowest values among all the models came from MP-1 and MP-5, respectively. The *DF*s of all the models were obtained according to Equation (3): 2.14, 1.46, 2.67, 2.61, 2.69, 2.22, 2.35, and 1.75 (Figure 11). With the same nitinol, the stents covered with PET grafts had higher *DF*s than those covered with e-PTFE grafts, which agreed with the existing research [30]. There was no obvious influence of graft materials on the fatigue performance of the SGs (*p* > 0.05), except in the group where the stents were made of nitinol-2. In addition, the properties of nitinol significantly affected the fatigue performance, especially in the e-PTFE group. For example, the *DF* of MP-2 was about 56% of the *DF* of MP-4. Therefore, the fatigue performance of the SGs was influenced by both the nitinol and graft.

Not only in terms of flexibility, the PET graft was also superior in the comparison of fatigue performance with the e-PTFE graft. Although the *DF*s of all SGs in the PET and e-PTFE groups were greater than 1.0, SGs in the PET group were safer since all the *DF*s exceeded 2. Finally, the effect of graft material on the SGs’ crimpability can be neglected, according to Figure 8 and Figure 9. Similar results can be found in Kleinstreuer C. et al. (2008) [30]. Therefore, PET is the preferred material in the design of SGs where flexibility and fatigue performance are the issues of concern.

The influence of nitinol on the fatigue performance was obvious through the analysis of *DF*s in the PET and e-PTFE groups. However, the trends in the *DF* and stiffness in both austenitic and martensitic crystals were not consistent, and it was difficult to confirm their relationships based on the alternate strain fields. Similar to the flexibility, the Poisson’s ratio was found to affect the fatigue performance of SGs through a comparison of the *DF*s obtained from the nitninol-3 and nitinol-4 stents. In future studies, microscopic structural analyses of nitinol may help reveal the mechanisms influencing its material properties.

## 4. Conclusions

This study systematically explored the effects of material properties on the mechanical performance of aortic arch SGs through finite element analysis of eight configurations combining four nitinol stents with PET or e-PTFE grafts. The key findings aligned with the research goal of clarifying material-driven optimization pathways are as follows:

1. The material properties of both nitinol and grafts significantly influence SGs’ core mechanical properties. Specifically, PET grafts outperform e-PTFE in enhancing flexibility and fatigue performance.
2. The graft material shows a negligible impact on crimpability, as the radial force and equivalent stress during compression are primarily determined by nitinol’s properties.
3. Nitinol’s properties dominate all performance metrics. A lower austenitic Young’s modulus (nitinol-3/4) improves flexibility. Nitinol-2 has higher radial force; nitinol-3/4 exhibit lower forces, influenced by phase transformation. Fatigue resistance depends on the nitinol-graft pairing, with nitinol-3 + PET (MP-5) showing optimal durability.
4. These results confirm the potential of material science-driven optimization: tailoring nitinol’s composition and manufacturing processes to adjust the initial transformation stress and strain can improve flexibility and crimpability, while selecting PET grafts is preferable for applications prioritizing flexibility and fatigue resistance.

## Figures and Tables

**Table 1:** Main parameters of four nitinols [21,30].

	Nitinol-1	Nitinol-2	Nitinol-3	Nitinol-4
Austenite Young’s modulus *E_A_* (MPa)	60,000	51,700	40,000	40,000
Austenite Poisson’s ratio $\mu_{A}$	0.33	0.3	0.33	0.46
Martensite Young’s modulus *E_M_* (MPa)	40,000	47,800	32,000	18,554
Martensite Poisson’s ratio $\mu_{M}$	0.33	0.3	0.33	0.46
Transformation strain $\varepsilon^{L}$	0.041	0.063	0.041	0.04
Loading δσδTLMPaT−1	6.7	6.527	6.7	6.527
Start of transformation loading σSMPatL	520	600	440	390
End of transformation loading σEMPatL	540	670	540	425
Reference temperature T_0_ (°C)	22	37	22	37
Unloading δσδTUMPaT−1	6.7	6.527	6.7	6.527
Start of transformation loading σSMPatU	250	288	250	140
End of transformation loading σEMPatU	140	254	140	135
Strain limit $\varepsilon_{\max}$	12%	12%	12%	12%

**Table 2:** Material properties of expanded polytetrafluoroethylene (e-PTFE)/polyethylene terephthalate (PET) [14,30].

	Elastic Modulus (MPa)	Poisson’s Ratio	Yield Stress (MPa)	Tensile Strength	Graft Thickness (mm)
e-PTFE	55.2	0.46	6.6	8.0	0.1
PET	1.84	0.35	59.3	86	0.1

**Table 3:** Composition of each model.

	Nitinol-1	Nitinol-2	Nitinol-3	Nitinol-4
PET	MP-1	MP-3	MP-5	MP-7
e-PTFE	MP-2	MP-4	MP-6	MP-8

## Data Availability

The original contributions presented in the study are included in the article, further inquiries can be directed to the corresponding author.
